# Effects of Temperature on Growth, Pathogenicity, and Fungicide Sensitivity of Two *Coniella* Species Causing Grape White Rot

**DOI:** 10.3390/jof12070535

**Published:** 2026-07-20

**Authors:** Lifang Yuan, Bo Liu, Tinggang Li, Xiangtian Yin, Qibao Liu, Xilong Jiang, Xing Han, Chundong Wang, Tao Ji, Nanyang Li, Yanfeng Wei

**Affiliations:** 1 Shandong Academy of Grape, Shandong Academy of Agricultural Sciences, Jinan 250100, China; ylifang1225@126.com (L.Y.);; 2 School of Landscape and Ecological Engineering, Hebei University of Engineering, Handan 056038, China; 3 Agricultural College, Shihezi University, Shihezi 832003, China; jitaoair@outlook.com

**Keywords:** *Coniella vitis*, *C. diplodiella*, temperature, growth, pathogenicity, virulence assay

## Abstract

Grape white rot, primarily caused by *Coniella diplodiella* and *Coniella vitis*, is a major fungal disease threatening grape production worldwide, with *C. vitis* more prevalent in China. Temperature is a critical environmental factor influencing pathogen development and disease outbreaks, but how temperature influences the biological characteristics, pathogenicity, and fungicide sensitivity of these two pathogens remains limited. This study aimed to evaluate the effects of temperature on mycelial growth, conidial germination, extracellular enzyme activity, pathogenicity, and fungicide sensitivity of *C. diplodiella* 2019 and *C. vitis* GP1. The growth, conidial germination, extracellular enzyme production, pathogenicity, and fungicide control efficacy against the two pathogens were systematically evaluated under different temperature conditions (5, 15, 25, 35, and 45 °C). Results showed temperature significantly affected all investigated traits in both species. Optimal mycelial growth, conidial germination, and pathogenicity occurred at moderate temperatures (25 °C), whereas extreme temperatures (5 °C and 45 °C) significantly markedly inhibited fungal development and disease severity. *C. vitis* GP1 showed greater adaptability and consistently higher pathogenicity than *C. diplodiella* 2019 across the tested temperature range. Fungicide sensitivity also differed between the two species, and the inhibitory activity of difenoconazole was significantly reduced under temperature extremes, indicating that fungicide performance is influenced by environmental temperature. These findings demonstrate that temperature regulates both fungal development and fungicide efficacy, providing new insights into the temperature-dependent epidemiology of grape white rot and a scientific basis for optimizing disease management under changing environmental conditions.

## 1. Introduction

Grape white rot is a destructive fungal disease widely distributed in grape-growing regions worldwide. The disease is most severe under warm and rainy climatic conditions, typically resulting in 20–30% yield losses and 30–50% economic losses annually, while severe epidemics can lead to complete crop failure [1,2]. The pathogen primarily infects leaves and mature fruits causing lesions that initially appear as light brown, water-soaked spots with irregular edges on the leaves. As infection progresses, black pycnidia were also observed on the infected leaves and berries; eventually, infected berries decay, shrivel, and become mummified [3,4,5,6].

Grape white rot is mainly caused by *C. diplodiella*, *C. fragariae*, *Pilidiella castaneicola*, and *C. vitis* [2,6,7]. In China, the disease was first reported in 1899, and *C. vitis* has since become the dominant pathogen [3,7]. The pathogenicity of fungal pathogens depends on their ability to colonize host tissues and overcome plant defense responses [8]. *C. vitis* infects a wide range of table and wine grape cultivars, including Sun Muscat, Red Globe, Kyoho, Cabernet Sauvignon, Merlot, and Xiaomangshen [9]. In addition, *C. vitis* produces secondary metabolites such as aflatrem and penitrem, which may pose risks to food safety and human health [8].

Temperature is a critical environmental factor influencing the growth, reproduction, and pathogenicity of plant pathogens, as well as the occurrence of plant diseases [10,11]. *Botrytis cinerea*, for example, infects most efficiently at 23 °C under high humidity, with disease incidence declining sharply above 30 °C [12]. Similarly, *C. diplodiella* exhibits optimal infection at 23.8 °C and peak sporulation at 20 °C [2,13]. Pathogenicity-related enzymes such as polygalacturonase, cellulase, and hemicellulase are also temperature sensitive, suggesting that thermal conditions may modulate virulence through enzymatic activity [4,14,15]. However, most studies have focused on optimal temperature ranges, leaving a key knowledge gap regarding pathogen responses in terms of survival, adaptation, and infectivity under extreme high temperatures (>35 °C), which are becoming more frequent due to climate change (IPCC 2023). Such extremes may not only suppress pathogen activity directly but also induce physiological stress responses to alter subsequent infectivity.

Chemical fungicides are widely used for controlling fungal fruit diseases and remain one of the fastest and most effective approaches for managing grape white rot [16]. Several fungicides, including phenamacril, flusilazole, pyraclostrobin, and tebuconazole, are commonly used in vineyards; however, the development of fungicide resistance has increasingly challenged disease management [17,18]. For example, *C. vitis* exhibits resistance to phenamacril and thiophanate-methyl, whereas fluazinam remains effective due to pathogen hypersensitivity [6]. Since repeated or exclusive reliance on fungicides accelerates resistance development, understanding the fungicide sensitivity of *C. diplodiella* and *C. vitis* is essential for optimizing chemical control strategies. Moreover, fungicide efficacy is closely associated with environmental temperature, which can influence pathogen metabolism, cell membrane stability, and detoxification processes, thereby affecting chemical sensitivity and disease control outcomes [19,20]. However, how temperature affects the biological characteristics, pathogenicity, and fungicide sensitivity of *C. diplodiella* and *C. vitis* remains largely unknown.

Therefore, this study aimed to systematically evaluate the effects of temperature on mycelial growth, conidial germination, extracellular enzyme activity, pathogenicity, and fungicide sensitivity of *C. diplodiella* 2019 and *C. vitis* GP1. Specifically, we investigated (i) the temperature-dependent biological and pathogenic responses of the two pathogens and (ii) the influence of temperature on fungicide sensitivity and control performance, with the goal of providing a scientific basis for developing temperature-adaptive management strategies for grape white rot.

## 2. Materials and Methods

### 2.1. Strains and Culture Conditions

In this study, representative strains of *C. diplodiella* 2019 and *C. vitis* GP1 were used. Both strains were originally obtained from symptomatic grape leaves showing typical white rot symptoms. *C. vitis* GP1 was deposited as a reference strain (CGMCC no. 23888) in the China General Microbiological Culture Collection Center and identified via whole-genome sequencing (GenBank: GCA_040437665.1) [9]. *C. diplodiella* 2019 was kindly provided by Professor Vittorio Rossi and was previously designated as strain COD2D. It was identified based on *tef1* gene sequencing and phylogenetic analysis [2] and is herein referred to as “2019” for consistency. They were preserved in 20% glycerol at −80 °C. For routine use, the cultures were grown on potato dextrose agar (PDA; Solarbio Life Sciences Co., Ltd., Beijing, China) or in potato dextrose broth (PDB) at 28 °C.

### 2.2. Field Temperature Monitoring

Ambient temperature in the experimental vineyard was continuously monitored using an internet-connected automatic weather station installed between grape rows at a height of approximately 1.5 m. Temperature data were recorded hourly throughout the grape-growing season.

### 2.3. Effects of Temperature on Mycelial Growth and Spore Germination

Mycelial plugs (4 mm diameter) from the margins of 3-day-old PDA cultures were placed onto the center of fresh PDA plates. Plates were incubated at 5, 15, 25, 35, and 45 °C in darkness; the colony diameters were measured on nine PDA plates at each temperature after 3 days of incubation, and the experiment was conducted three times.

For liquid culture, five mycelial plugs were inoculated into 150 mL of PDB in 250 mL Erlenmeyer flasks. Flasks were incubated at 180 rpm at the aforementioned temperatures. Biomass was determined by harvesting, drying (60 °C for 48 h), and weighing the mycelia after 7 days. Medium pH was recorded on days 1, 3, 5, and 7. The experiment was performed with five flasks per treatment and repeated three times.

Conidia were harvested from 14-day-old PDA cultures incubated at 28 °C. A suspension of 1 × 10^6^ conidia/mL in sterile distilled water was prepared. Aliquots (100 µL) were placed on sterile concave slides and incubated at 5, 15, 25, 35, and 45 °C. After 14 h, germination (germ tube length ≥ spore diameter) and germ tube length were assessed for at least 100 conidia per replicate under a light microscope. Five slides per treatment comprised one replicate, and the experiment was repeated three times.

### 2.4. Effects of Temperature on Cell Wall-Degrading Enzyme (CWDE) Activity

Conidial suspensions (1 × 10^6^ conidia/mL) were inoculated into 25 mL of synthetic mineral medium containing 0.2 g/L MgSO_4_·7H_2_O, 0.4 g/L KH_2_PO_4_, 0.2 g/L KCl, 1 g/L NH_4_NO_3_, 0.01 g/L FeSO_4_, 0.01 g/L ZnSO_4_, and 0.01 g/L MnSO_4_. The medium was supplemented with 1% (*w*/*v*) polygalacturonic acid (PGA) for polygalacturonase (PG) production or 1% (*w*/*v*) carboxymethyl cellulose (CMC) for cellulase (Cx) production. Cultures were incubated at 5, 15, 25, 35, and 45 °C on a rotary shaker at 200 rpm for 3 days. Culture filtrates were collected and used as crude enzyme extracts.

For PG activity, 100 µL of crude extract was added to wells on assay medium containing 10 g/L PGA, 10 g/L yeast extract, 2.2 mmol/L EDTA, and 110 mmol/L sodium acetate buffer (pH 5.5) with 20 g/L agarose. Plates were incubated at 30 °C for 18 h, and enzyme activity was visualized by flooding with 4 M HCl. The diameter of clear hydrolysis zones was measured.

For Cx activity, 100 µL of crude extract was added to wells on agar medium containing 1 g/L CMC and 25 mmol/L sodium phosphate buffer (pH 7.0) with 20 g/L agar. After incubation, plates were stained with 0.1% Congo red solution, and hydrolysis zones were measured after destaining.

All assays were performed with three technical replicates per sample and independently repeated twice using separate culture flasks for each temperature treatment. Data were expressed as mean ± standard deviation (SD).

### 2.5. Effects of Temperature on Pathogenicity

Detached leaves of *Vitis vinifera* ‘Red Globe’ were surface-disinfested with 75% ethanol. A 4 mm mycelial plug from a 3-day-old culture was placed onto each leaf. To maintain leaf turgor and prevent desiccation, both ends of the detached leaves were wrapped with moistened sterile cotton balls. The inoculated leaves were then placed on moistened sterile filter paper in plastic humidity chambers to maintain high humidity conditions (approximately 90% relative humidity, RH). Inoculated leaves were incubated at 5, 15, 25, 35, and 45 °C. Lesion diameter was measured after 3 days. Each treatment consisted of 10 leaves, and the experiment was performed three times.

### 2.6. In Vitro Fungicide Sensitivity Assays

The sensitivity of *C. diplodiella* 2019 and *C. vitis* GP1 to seven fungicides, including carbendazim (80% active ingredient; Qingdao Fengfei Biotechnology Co., Ltd., Qingdao, China), tebuconazole (98.2%; Shandong Lutong Biotechnology Co., Ltd., Jining, China), difenoconazole (96.7%; Qingdao Fengfei Biotechnology Co., Ltd., Qingdao, China), flusilazole (95.0%; Shandong Lutong Biotechnology Co., Ltd., Jining, China), iprodione (96.6%; Qingdao Fengfei Biotechnology Co., Ltd., Qingdao, China), pyrimethanil (98%; Hebei Mojin Biotechnology Co., Ltd., Shijiazhuang, China), and pyraclostrobin (98.0%; Shandong Lutong Biotechnology Co., Ltd., Jining, China), was evaluated using a mycelial growth inhibition assay. Mycelial plugs (4 mm in diameter) taken from 3-day-old cultures were placed onto PDA amended with each fungicide at concentrations of 5, 10, 50, 100, 500, and 1000 μg/mL. PDA plates without fungicide served as controls. All plates were incubated at 28 °C in the dark for 3 days. Each concentration was replicated five times, and the entire experiment was performed three times independently.

The inhibition rate was calculated as follows: Inhibition rate (%) = [(Colony diameter of control (mm) − Colony diameter of treatment (mm))/Colony diameter of control (mm)] × 100 [21,22].

Concentration–response data were analyzed by probit regression, with inhibition rate (Y) as the dependent variable and log-transformed fungicide concentration (X) as the independent variable.

The regression model Y = aX + b was used to estimate the median effective concentration (EC_50_) and the coefficient of determination (R^2^), where a and b represent the slope and intercept, respectively.

### 2.7. In Vitro Efficacy of Fungicides Against Grape White Rot

To evaluate the control efficacy of fungicides, fifteen grape berries were inoculated with 10 µL suspensions of *C. diplodiella* 2019 and *C. vitis* GP1 (1 × 10^6^ spores/mL), respectively. After 24 h, seven fungicides (carbendazim, tebuconazole, difenoconazole, flusilazole, iprodione, pyrimethanil and pyraclostrobin) of 50 mg/L were sprayed on the grape berries, respectively. Treated berries were kept at 28 °C and 100% RH. Lesion diameter and disease incidence were measured after 3 days.

Disease incidence = number of diseased grape berries/number of total investigated grape berries × 100%

### 2.8. In Vivo Efficacy of Fungicides Against Grape White Rot

Five 1-year-old *V. vinifera* cv. Red Globe plants were sprayed with spore suspensions (1 × 10^6^ spores/mL) of *C. diplodiella* 2019 or *C. vitis* GP1. After 24 h, tebuconazole, difenoconazole and flusilazole were applied for curative assays. Plants were maintained at 28 °C and 100% RH. Three days after inoculation, the disease index and control efficacy were calculated as described by Yang et al. [22].

Disease severity on grape leaves was rated on a 0–9 scale based on the percentage of diseased leaf area: 0, no symptoms; 1, 0–5%; 3, 6–25%; 5, 26–50%; 7, 51–75%; 9, 76–100%. Disease index (DI) was calculated as DI = [(∑disease grades × number of infected leaves)/(total checked leaves × 9)] × 100.

Control efficacy = (DI for the control − DI for the fungicides)/DI for the control × 100% [22].

### 2.9. Cross-Resistance Analysis Among Fungicides

Seven field isolates of *C. vitis* were used to evaluate potential cross-resistance among flusilazole, difenoconazole, pyrimethanil, iprodione, and carbendazim. The sensitivity of each isolate to each fungicide was determined by calculating EC_50_ values using the mycelial growth inhibition method as described in Section 2.6.

To assess cross-resistance, Spearman’s rank correlation coefficients were calculated based on the log_10_-transformed EC_50_ values between all pairwise combinations of fungicides [23,24]. A significant positive correlation was interpreted as potential evidence of cross-resistance between fungicides.

### 2.10. In Vivo Effect of Environmental Temperature on Fungicide Efficacy

To assess the effect of temperature on fungicide performance, ten grape berries were inoculated by pipetting 10 μL of spore suspensions of *C. vitis* GP1 and *C. diplodiella* 2019 (1 × 10^6^ spores/mL), respectively; then, flusilazole and difenoconazole (50 mg/L) were applied. Treated berries were kept at 5, 15, 25, 35 and 45 °C for 2 h and then transferred to a greenhouse at 28 °C and 100% RH.

DI and control efficacy were evaluated 3 days after inoculation, following the method of Yin [25] and Yuan [9]. Disease severity on detached grape fruits was assessed based on the percentage of symptomatic surface area using a 0–4 rating scale: 0 = no symptoms; 1 = ≤25% of fruit surface area diseased; 2 = 26–50% diseased; 3 = 51–75% diseased; 4 = >75% diseased. The DI was calculated as DI = [(∑disease grades × number of infected fruits)/(total checked fruits × 4)] × 100.

Control efficacy = (DI for the control − DI for the fungicides)/DI for the control × 100% [22].

### 2.11. Statistical Analysis

Experimental data were statistically analyzed using SPSS 22.0 software. One-way ANOVA followed by the LSD test was performed to assess differences between temperatures, and efficacy of fungicides; *p* values < 0.05 were considered statistically significant.

## 3. Results

### 3.1. Field Temperature Conditions During the Grape Growing Season

**Figure 1:**
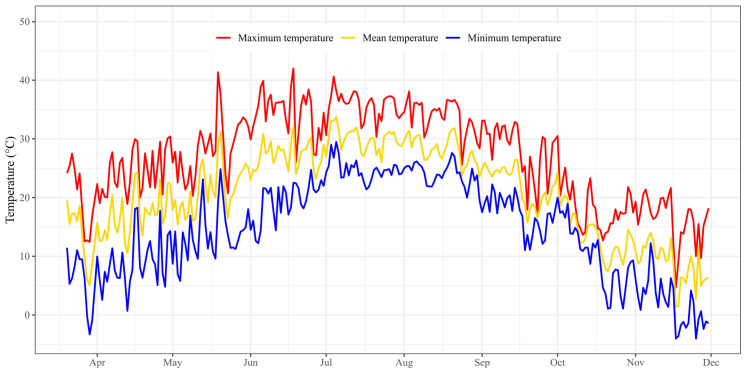
Field temperature dynamics during the grape growing season.

Field temperature was monitored in a vineyard in the Jiyang District of Jinan, Shandong Province, from March to November in 2024. During this period, daily mean temperatures ranged from 5.07 to 33.73 °C, with minimum and maximum temperatures ranging from −4.02 to 41.99 °C. Approximately 90% of the daily mean temperatures fell within 5–35 °C, which aligns well with the lower five temperature treatments (5, 15, 25, 35 °C) used in laboratory assays (Figure 1). For temperatures above 35 °C, which occurred on 52 days (with three days exceeding 40 °C), the laboratory design included a 45 °C treatment to capture the physiological responses to extreme heat events.

### 3.2. Effect of Temperature on Mycelial Growth and Spore Germination

**Figure 2:**
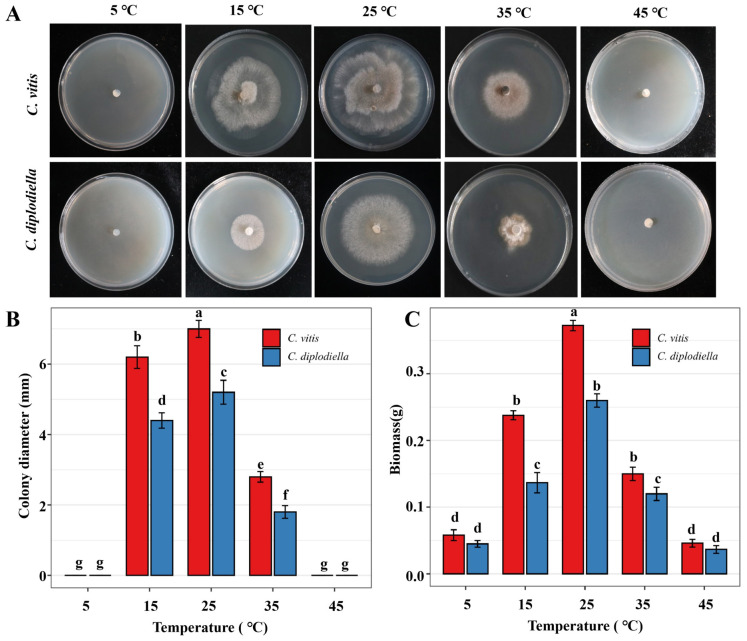
The growth of *C. diplodiella* 2019 and *C. vitis* GP1 at different temperatures: (**A**) Colony morphology of *C. diplodiella* 2019 and *C. vitis* GP1. (**B**) The colony diameter of *C. diplodiella* 2019 and *C. vitis* GP1 cultured at different temperatures. (**C**) The mycelial dry weight of *C. diplodiella* 2019 and *C. vitis* GP1 cultured in PDB media for 3 days at different temperatures. Different lowercase letters above the bars indicate significant differences among treatments (*p* < 0.05).

Laboratory assays showed that mycelial growth of both *C. vitis* GP1 and *C. diplodiella* 2019 was optimal at 25 °C (Figure 2A). Growth was significantly slower at 15 °C and 35 °C and completely inhibited at 5 °C and 45 °C. Dry weight measurements in PDB confirmed that biomass accumulation was maximal at 25 °C (0.37 g for *C. vitis* GP1 and 0.26 g for *C. diplodiella* 2019), while negligible mycelium was obtained at 5 °C and 45 °C (Figure 2B,C). Notably, *C. vitis* GP1 exhibited faster growth than *C. diplodiella* 2019 at all tested temperatures, resulting in larger colony diameters and greater biomass accumulation for the former.

**Figure 3:**
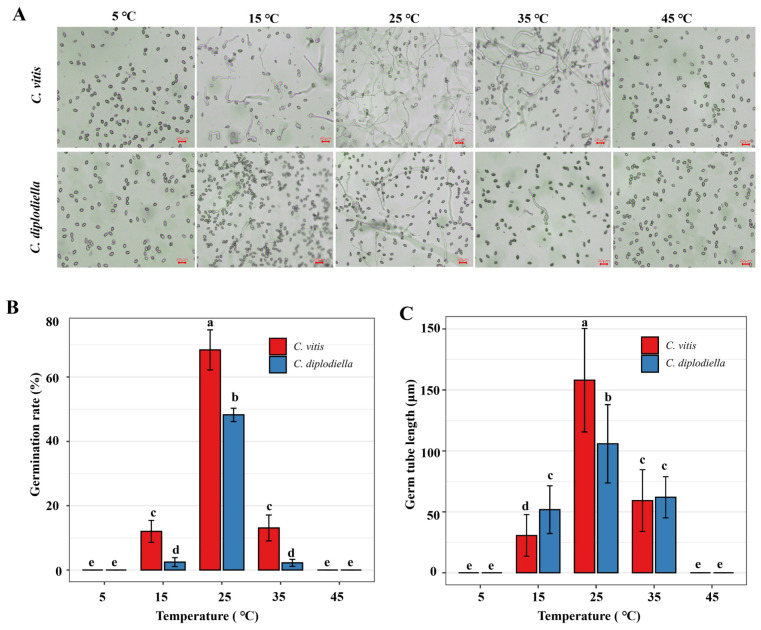
Microscopic morphology of spore germination of *C. diplodiella* 2019 and *C. vitis* GP1 under different temperatures: (**A**) Scale bars = 20 μm. Germination rate (**B**) and germ tube length of *C. diplodiella* 2019 and *C. vitis* GP1 (**C**). Different lowercase letters above the bars indicate significant differences among treatments (*p* < 0.05).

The spores of *C. diplodiella* 2019 and *C. vitis* GP1 did not germinate at 5 °C and 45 °C, and spore germination was observed within the temperature range of 15–35 °C for both fungi, with the highest germination rates occurring at 25 °C (Figure 3A). A higher germination rate was observed for *C. vitis* GP1 compared to *C. diplodiella* 2019 at 15–35 °C; for example, the germination rates of *C. diplodiella* 2019 and *C. vitis* GP1 are 48.25% and 68.46% at 25 °C, respectively (Figure 3B,C). However, germination rates declined at higher temperatures, and the germination rates of *C. diplodiella* 2019 and *C. vitis* GP1 decreased by 11.34% and 54.91% at 35 °C, respectively (Figure 3B,C).

### 3.3. Effect of Temperature on pH Value and Enzyme Activity of C. diplodiella 2019 and C. vitis GP1

**Figure 4:**
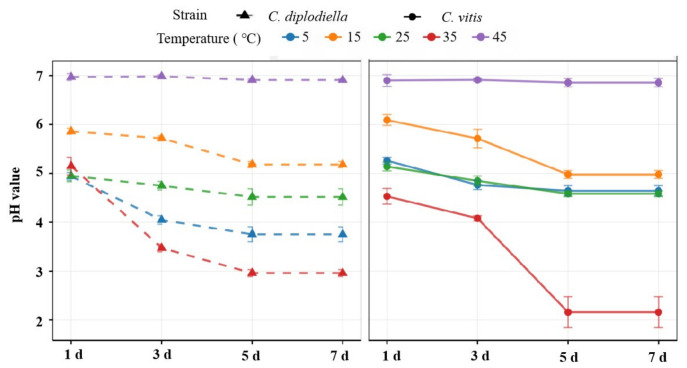
Variation in pH in PDB during the growth of *C. diplodiella* 2019 and *C. vitis* GP1.

The pH of PDB medium declined during the cultivation of *C. diplodiella* 2019 and *C. vitis* GP1 between 15 and 35 °C, with more substantial acidification occurring at higher temperatures (Figure 4). At 35 °C, the pH decreased from 4.66 to 1.71 for *C. diplodiella* 2019 and from 5.12 to 1.96 for *C. vitis* GP1. In contrast, pH remained essentially unchanged in cultures maintained at 45 °C.

**Figure 5:**
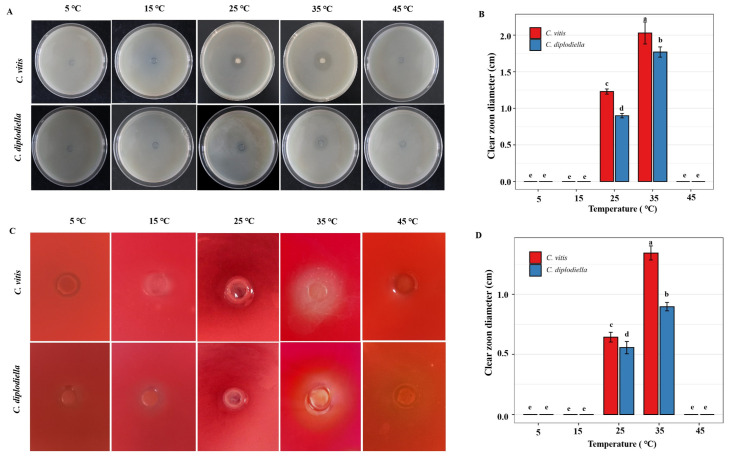
Polygalacturonase (**A**,**B**) and cellulase (**C**,**D**) and polygalacturonase activity produced by *C. diplodiella* 2019 and *C. vitis* GP1 at different temperatures. Different lowercase letters above the bars indicate significant differences among treatments (*p* < 0.05).

Temperature significantly influenced the production of plant CWDEs by both fungi. Polygalacturonase and cellulase activities were undetectable at 5, 15, or 45 °C (Figure 5A,C). Notably, within the range of 25 to 35 °C, the diameters of the enzymatic hydrolysis zones for both enzymes increased with rising temperature, indicating enhanced secretion of polygalacturonase and cellulase (Figure 5B,D). Furthermore, the enzymatic hydrolysis zones produced by *C. vitis* GP1 were consistently larger than those of *C. diplodiella* 2019 at these temperatures (Figure 5B,D).

### 3.4. Effect of Temperature on the Pathogenicity of C. diplodiella 2019 and C. vitis GP1

**Figure 6:**
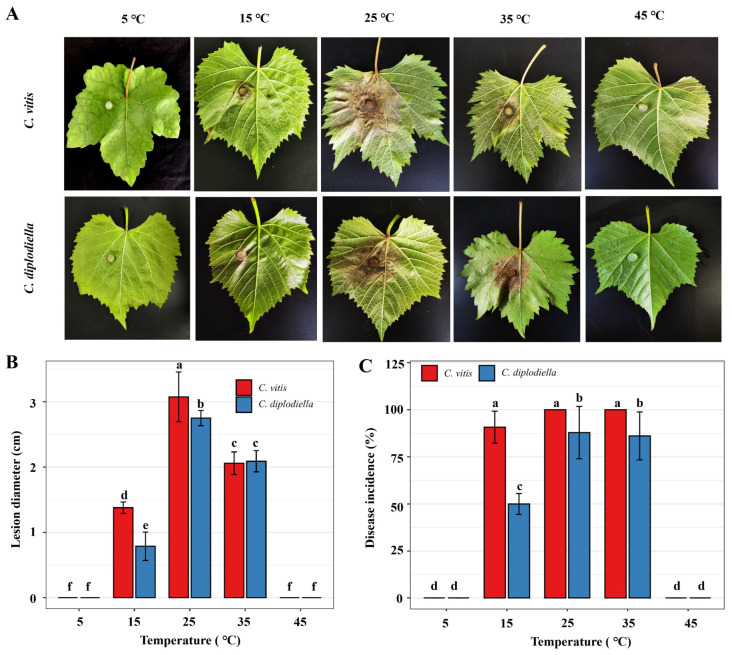
The pathogenicity of *C. diplodiella* 2019 and *C. vitis* GP1 at different temperatures: (**A**) Symptoms on grape leaves of inoculation with *C. diplodiella* 2019 and *C. vitis* GP1 for 3 days at different temperatures. (**B**) The diameters of lesions on grape leaves. (**C**) The incidence of grape white rot on leaves inoculated with *C. diplodiella* 2019 and *C. vitis* GP1 for 3 days at different temperatures. Different lowercase letters above the bars indicate significant differences among treatments (*p* < 0.05).

The pathogenic fungi *C. diplodiella* 2019 and *C. vitis* GP1 can cause grape white rot at temperatures between 15 and 35 °C; the highest pathogenicity was observed at 25 °C. Both fungi lost their ability to cause disease at 5 °C and 45 °C (Figure 6A). At these temperatures, the lesion diameters and disease incidence on grape leaves caused by *C. vitis* GP1 were higher than those caused by *C. diplodiella* 2019 in the temperature range of 15–35 °C (Figure 6B,C).

### 3.5. In Vitro Toxicity and Control Efficacy of Fungicides Against Grape White Rot Pathogens

Sensitivity of both pathogens to seven fungicides was determined under laboratory conditions (Table 1). *C. vitis* GP1 was highly sensitive to flusilazole and difenoconazole but resistant to tebuconazole. *C. diplodiella* 2019 was sensitive to carbendazim, difenoconazole, and flusilazole but had reduced susceptibility to tebuconazole and pyraclostrobin. For *C. vitis* GP1, conidial germination was not affected by difenoconazole, flusilazole, pyrimethanil, or iprodione at concentrations of 5–10 μg/mL; however, germ tube elongation was significantly inhibited, particularly by iprodione. At concentrations above 50 μg/mL, conidial germination was completely suppressed. In contrast, *C. diplodiella* 2019 was highly sensitive to pyrimethanil and iprodione, with germination completely inhibited at concentrations above 10 μg/mL. Under flusilazole treatment (5–50 μg/mL), conidial germination rates of *C. diplodiella* 2019 remained largely unchanged, but germ tube length was significantly reduced. Difenoconazole affected both germination and germ tube elongation of *C. diplodiella* 2019, with significant inhibition observed even at 5 μg/mL (Table S1).

**Figure 7:**
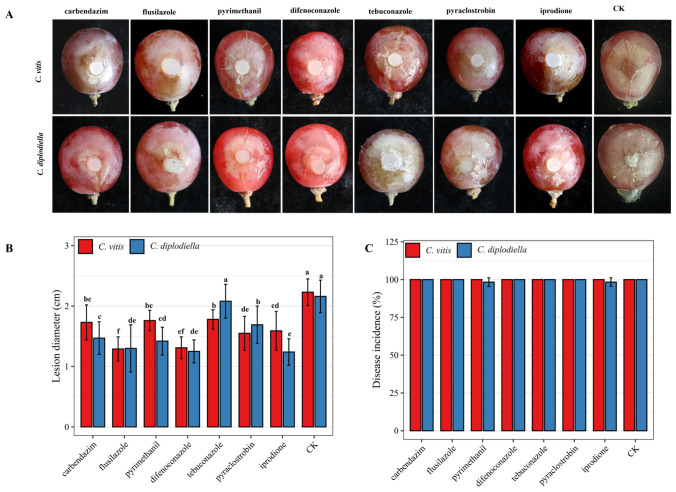
Inhibitory effects of different fungicides on grape white rot: (**A**) Disease symptoms, (**B**) lesion diameter (cm) and (**C**) disease incidence (%) of grape berries after treatment with different fungicides. Different lowercase letters above bars indicate significant differences among treatments within the same pathogen (*p* < 0.05). CK: Control (untreated).

Results from the detached fruit assay indicated distinct fungicide efficacy patterns between the two species. Flusilazole and difenoconazole were the most effective fungicides against *C. vitis* GP1; tebuconazole provided no control efficacy. Against *C. diplodiella* 2019, flusilazole, difenoconazole, and iprodione showed comparable efficacy, while tebuconazole and pyraclostrobin notably failed to provide effective control (Figure 7).

### 3.6. Efficacy of Flusilazole and Difenoconazole in Pot Experiments

Since both *C. diplodiella* 2019 and *C. vitis* GP1 were sensitive to flusilazole and difenoconazole, and resistant to tebuconazole, flusilazole and difenoconazole were selected to evaluate their control efficacy against grape white rot, with tebuconazole and sterile water serving as the fungicide control and negative control, respectively. The results demonstrated that flusilazole and difenoconazole exhibited strong control efficacy against *C. vitis* GP1 in potted plants, with efficacy exceeding 65%, and against *C. diplodiella* 2019, where efficacy surpassed 70%. In contrast, tebuconazole showed lower control efficacy, achieving 45.41% against *C. diplodiella* 2019 and only 18.19% against *C. vitis* GP1 (Table 2).

### 3.7. Cross-Resistance of Flusilazole and Difenoconazole with Other Fungicides

**Figure 8:**
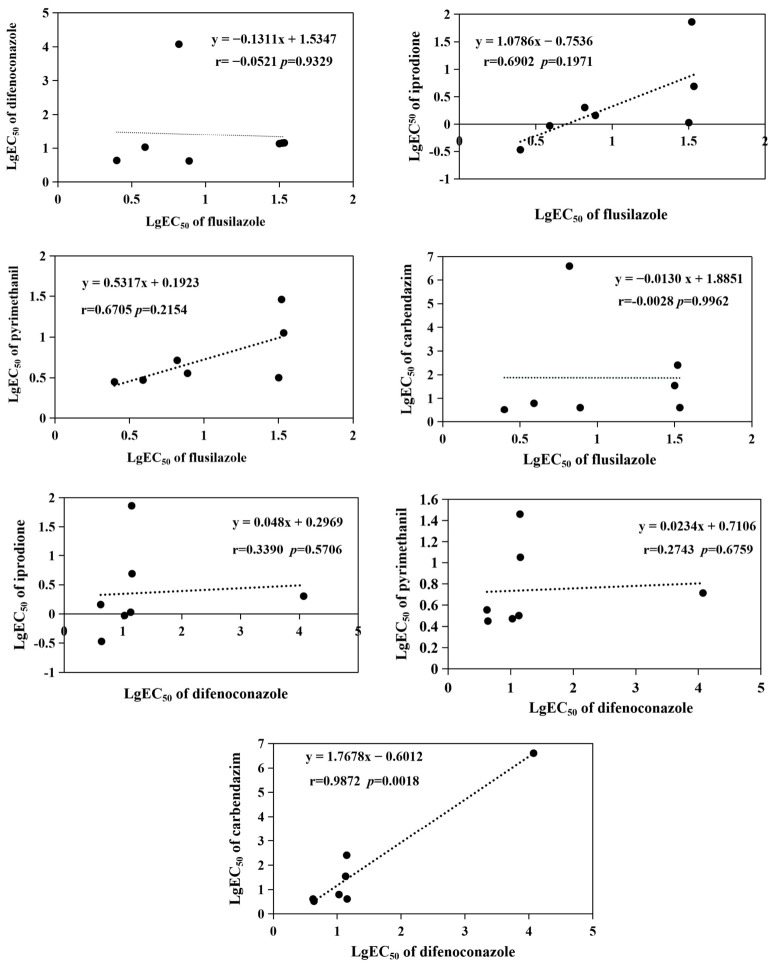
Cross-resistance of the selected isolates among different fungicides. The dashed lines represent the linear regression fitting lines.

To determine the cross-resistance between flusilazole/difenoconazole and other fungicides, the EC_50_ values of representative isolates were measured for each fungicide. A very weak negative correlation was observed between the resistance levels of flusilazole and either difenoconazole or carbendazim, but the relationships were not statistically significant, indicating no cross-resistance between these fungicides. Although the correlation coefficients between flusilazole (or difenoconazole) and iprodione or pyrimethanil suggested a positive relationship, the correlations were not statistically significant, showing no clear evidence of cross-resistance. Notably, difenoconazole and carbendazim showed a significant positive relationship (*p* < 0.05), alternating use of difenoconazole and carbendazim should be avoided (Figure 8).

### 3.8. Effect of Application Temperature on Fungicide Efficacy

**Figure 9:**
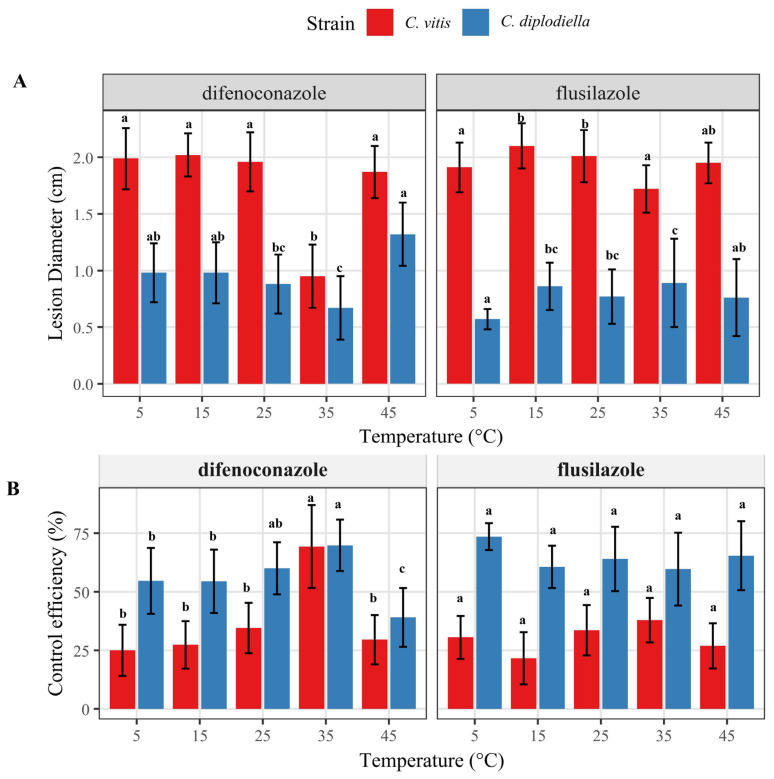
Effect of temperature on the efficacy of flusilazole and difenoconazole against *C. diplodiella* 2019 and *C. vitis* GP1: (**A**) Lesion diameter (cm) and (**B**) control efficiency (%) of grape berries after treatment with different fungicides at different temperatures. Different lowercase letters above bars indicate significant differences among treatments within the same pathogen (*p* < 0.05).

Temperature could influence the efficacy of fungicides, whether applied in the greenhouse or in the field. In this study, we found flusilazole treatment did not result in significant differences in lesion size among the temperature treatments tested, and the efficacy was not affected by the temperature (Figure 9). Under difenoconazole treatment, lesion diameter varied among temperature treatments, with the smallest lesions observed at 35 °C, and the efficacy was highest at this temperature (Figure 9).

## 4. Discussion

Grape white rot has emerged as a major constraint on grape production in China and other viticultural regions, particularly in wine grapes, where severe outbreaks can result in substantial yield and economic losses [26,27,28]. Many grape varieties, including both wine grapes (e.g., Cabernet Sauvignon, Petit Manseng, Merlot) and table grapes (e.g., Golden Finger, Kyoho, Red Globe), are highly susceptible to grape white rot [29]. Recent increases in disease incidence in table grapes, including cultivars such as Shine Muscat that were previously considered relatively tolerant, further highlight the expanding threat of this disease [8]. *C. vitis* has become the dominant pathogen of grape white rot in China. Our study provides a possible explanation: compared to the *C. diplodiella* 2019, the *C. vitis* GP1 exhibited a higher growth rate, greater spore germination, and stronger virulence, which may have contributed to this shift in pathogen prevalence.

Temperature is a critical environmental factor that profoundly influences fungal adaptability, pathogenicity, and evolutionary dynamics [30,31,32]. Grape white rot is prevalent in warm and humid regions, and previous studies have demonstrated that disease outbreaks caused by *C. diplodiella* are most favorable at 20–30 °C, while occurrence is rare below 12 °C or above 33 °C [2,33]. In this study, both *C. diplodiella* 2019 and *C. vitis* GP1 exhibited optimal mycelial growth and spore germination at 25 °C among the tested temperatures, whereas growth rates, spore germination, and infection efficiency were markedly reduced at temperatures below 15 °C or above 35 °C. Correspondingly, the latent period was significantly shortened within the range of 20–35 °C, particularly in wounded fruits, but was substantially prolonged at 10–15 °C. These results are consistent with findings in other pathosystems, such as *Colletotrichum acutatum* infecting olive, where the shortest latent period and highest disease severity were observed at 17–20 °C [34], and *Podosphaera clandestina* causing hawthorn powdery mildew, which showed an optimal latent period at approximately 23 °C with considerable prolongation at cooler temperatures [35]. Collectively, these findings highlight that temperature plays a decisive role in the epidemiology of grape white rot, and that extension of the incubation period under suboptimal conditions may mitigate epidemic risk of the disease. However, because the temperature intervals evaluated in this study were relatively broad, further investigations using more refined temperature gradients are needed to determine the precise optimum temperature range for growth, infection, and disease development. Moreover, our comparative analysis revealed that *C. vitis* GP1 exhibited faster growth, higher spore germination rates, and stronger virulence than *C. diplodiella* 2019 under favorable temperatures. This difference in pathogenic fitness may explain why *C. vitis* has gradually replaced *C. diplodiella* as the dominant causal agent of grape white rot in China.

Fungicides remain a cornerstone in the management of fungal diseases; however, studies addressing their efficacy against grape white rot have been relatively limited. A previous study reported that *C. vitis* was naturally resistant to phenamacril and thiophanate-methyl but remained susceptible to several other fungicides, including fluazinam, fludioxonil, difenoconazole, tebuconazole, pydiflumetofen, and pyraclostrobin [6]. In contrast, our results demonstrated that both *C. diplodiella* 2019 and *C. vitis* GP1 exhibited natural resistance to tebuconazole. Furthermore, the EC_50_ value of *C. vitis* GP1 for difenoconazole was nearly 100-fold higher than previously reported [6], indicating a potential shift in sensitivity and raising concern for emerging resistance to this fungicide. Among tested fungicides, flusilazole, a sterol demethylation inhibitor (DMI) that disrupts ergosterol biosynthesis and compromises fungal cell membrane integrity, showed the highest efficacy. This compound has been extensively applied in the control of apple, pear, cucumber, and tomato diseases [36,37,38]. In the present study, both *C. diplodiella* 2019 and *C. vitis* GP1 were highly sensitive to flusilazole and difenoconazole, and the strong performance in pot experiments further supports their potential as a promising option for managing grape white rot. Notably, the efficacy of difenoconazole was reduced under extremely high-temperature conditions (45 °C), which may be associated with temperature-related changes, such as reduced fungicide stability, altered cuticle penetration, or decreased retention on the fruit surface. However, the direct effects of temperature on fungicide properties were not evaluated in this study and require further investigation. Overall, these findings broaden current understanding of fungicide sensitivity profiles in grape white rot pathogens and provide a foundation for developing more effective and sustainable disease management strategies.

The long-term use of a single fungicide type frequently accelerates the development of resistance in pathogenic fungi, thereby compromising disease management [39,40,41]. Moreover, increasing fungicide dosages not only intensifies the risk of resistance but also raises concerns regarding environmental pollution and pesticide residues [42,43,44]. Consequently, the identification of alternative fungicides and complementary control strategies is critical for the sustainable management of grape white rot. In this study, no cross-resistance was detected between flusilazole (or difenoconazole) and iprodione, or pyrimethanil, suggesting that flusilazole and difenoconazole could be applied in alternation or combination with these fungicides to achieve more effective and durable control. Additionally, the biological control agents *Bacillus velezensis* GSBZ09 and *Paenibacillus peoriae* ZBSF16 significantly reduced disease severity in detached leaf and fruit assays [23,45]. Taken together, these results suggest that integrating chemical fungicides such as flusilazole and difenoconazole with biological control agents or other complementary strategies may reduce fungicide inputs, delay resistance development, and provide more stable and sustainable control of grape white rot across diverse environmental conditions. It should be noted that the direct effect of temperature on the fungicide itself was not assessed in this study; therefore, this possibility cannot be excluded and warrants further investigation.

## 5. Conclusions

This study highlights temperature as a key environmental factor shaping the epidemiological characteristics and chemical management of grape white rot caused by *C. diplodiella* and *C. vitis*. Rather than acting solely as a driver of pathogen growth, temperature influences multiple disease-related processes, including pathogen development, infection potential, and fungicide performance, thereby affecting overall disease risk and control outcomes. The distinct responses of the two *Coniella* species to temperature and fungicides indicate that disease management strategies should consider pathogen-specific characteristics rather than relying on a uniform control approach. These findings improve our understanding of temperature-dependent disease development and provide a basis for integrating environmental conditions into disease forecasting and fungicide application decisions for more precise and sustainable management of grape white rot.

## Figures and Tables

**Table 1:** Toxicity determination of different chemical agents against grape white rot pathogens *C. diplodiella* 2019 and *C. vitis* GP1.

Strain	Fungicide	Toxicity Equation	Correlation Coefficient R2	EC50 (μg/mL)
*C. vitis* GP1	carbendazim	Y = 1.3259x + 1.9548	0.923	204.570
	tebuconazole	Y = 0.3775x + 2.7948	0.824	/
	difenoconazole	Y = 1.3879x + 2.4588	0.867	67.759
	flusilazole	Y = 0.9359x + 3.716	0.949	23.547
	pyraclostrobin	Y = 1.9353x + 0.6202	0.867	183.279
	pyrimethanil	Y = 1.9459x + 2.1586	0.968	28.840
	iprodione	Y = 1.3887x + 2.4216	0.903	72.440
*C. diplodiella* 2019	carbendazim	Y = 1.5355x + 2.344	0.923	53.669
	tebuconazole	Y = 0.1188x + 4.2955	0.816	/
	difenoconazole	Y = 1.4388x + 2.3153	0.981	73.439
	flusilazole	Y = 1.5862x + 2.5615	0.738	34.460
	pyraclostrobin	Y = 0.9836x + 1.9442	0.903	/
	pyrimethanil	Y = 2.6866x + 7.7017	0.991	0.099
	iprodione	Y = 2.8919x + 8.7394	0.977	0.051

Note: “/” indicates no EC_50_ value could be obtained within the tested concentration range.

**Table 2:** Effect of different fungicides on grape white rot caused by *C. diplodiella* 2019 and *C. vitis* GP1.

Fungicide	*C. vitis* GP1			*C. diplodiella* 2019		
	Disease Index	Incidence (%)	Control Efficacy (%)	Disease Index	Incidence (%)	Control Efficacy (%)
tebuconazole	50.15 ± 6.80 b	81.64 ± 8.07 b	38.68 ± 6.57 b	42.11 ± 4.89 b	91.32 ± 3.63 b	45.41 ± 2.88 c
difenoconazole	30.02 ± 6.43 c	72.22 ± 5.98 c	65.28 ± 6.25 a	17.75 ± 6.18 c	74.46 ± 5.91 c	73.03 ± 3.16 b
flusilazole	23.25 ± 2.10 c	66.68 ± 7.41 c	77.27 ± 5.93 a	14.7 ± 5.23 c	60.55 ± 7.23 c	80.94 ± 5.40 a
CK	88.58 ± 6.36 a	97.22 ± 1.02 a	—	77.15 ± 5.15 a	100 a	—

Note: Different lowercase letters within the same column indicate significant differences at *p* < 0.05.

## Data Availability

Data are contained within the article and Supplementary Materials.

## Supplementary Materials

The following supporting information can be downloaded at: https://www.mdpi.com/article/10.3390/jof12070535/s1, Table S1: Effects of different fungicides on conidial germination and germ tube elongation of *C. vitis* GP1 and *C. diplodiella* 2019.
