# Supplementary material for: Effects of Temperature on Growth, Pathogenicity, and Fungicide Sensitivity of Two Coniella Species Causing Grape White Rot
Source: J Fungi (Basel). 2026 Jul 20;12(7):535. doi: 10.3390/jof12070535 (PMC13412259; doi:10.3390/jof12070535)
Supplement: Supplementary file 1 [file jof-12-00535-s001.zip › jof-4398068-supplementary.pdf]

Table S1. Effects of different fungicides on conidial germination and germ tube elongation of *C. vitis* GP1and *C. diplodiella* 2019.

Strain	Concentration (μg/mL)	difenoconazole	flusilazole	pyrimethanil	iprodione
C. *vitis*	0	187.29±31.39 (92.14±18.85)	192.88±39.03 (91.26±13.94)	220.66±14.42 (93.28±10.87)	165.59±18.04 (92.32±12.47)
	5	134.04±27.24 (90.45±15.23)	117.35±14.06 (88.13±11.44)	215.58±19.40 (85.93±13.94)	14.08±2.59 (90.26±15.87)
	10	44.30±7.97 (84.98±16.67)	63.25±10.60 (86.81±15.33)	144.90±18.65 (87.38±15.17)	9.25±1.73 (85.56±14.67)
	50	/	/	35.78±6.18 (81.13±12.47)	/
	100	/	/	/	/
	500	/	/	/	/
	1000	/	/	/	/
C. diplodiella	0	256.27±56.92 (91.23±12.32)	250.83±30.84 (90.34±13.17)	237.40±32.57 (89.23±11.32)	279.53±81.62 (94.23±17.16)
	5	123.77±8.22 (89.23±13.46)	132.15±27.48 (88.97±15.25)	16.59±5.11 (87.57±14.32)	/
	10	62.20±14.42 (86.74±11.84)	38.32±8.42 (89.45±12.36)	/	/
	50	22.75±6.64 (40.97±16.65)	14.45±5.25 (86.49±14.68)	/	/
	100	21.51±10.14 (3.75±2.17)	/	/	/
	500	/	/	/	/
	1000	/	/	/	/

Note: Data represent germ tube length (μm, upper row) and germination rate (%, lower row in parentheses) (mean ± SD). The symbol "/" indicates complete inhibition of both germination and germ tube elongation at the tested concentration.
